# An Essay upon Clasps for Retaining Partial Sets of Artificial Teeth in the Mouth

**Published:** 1851-07

**Authors:** Charles A. Dubouchet


### ARTICLE XII.

*An Essay upon Clasps for Retaining Partial Sets of Artificial Teeth in the Mouth.* By Charles A. Dubouchet, M. D. Read before the Pennsylvania Association of Dental Surgeons, February 7th, 1851.

Mr. President—In accordance with the rules of this society, it has been your pleasure to appoint me to prepare an Essay on some subject relating to our profession. That much good may be accomplished by following up this method, every member of this society must feel convinced.

The high tone of improvement pervading the dental community demands at our hands a continuance of efforts to keep in the onward track.

The "Dental News Letter" affords us such an excellent channel to diffuse throughout the land whatever information we may have to impart, that we should not neglect to improve the opportunity. The papers published in it upon "Filling Teeth," and "Mechanical Dentistry," as well as upon other subjects, contributed by our members, have shed upon the Pennsylvania Society of Dental Surgeons a lustre, in the bright effulgence of which any dentist may *well* feel proud to bask.

Under such circumstances, the task allotted me becomes important, and such precedents render me conscious of inability. Still, Mr. President, deeming it a duty and an honor, and relying upon the indulgence and proverbial kindness of our members, I shall proceed to make a few remarks.

Lately great improvements have been made in mechanical dentistry. First in the rank we notice atmospheric plates, used for the insertion of one or two teeth, in the place of the old-fashioned plates with clasps; and our fellow member, Dr. White, is among the first to have given his attention to this new mode of insertion.

This seems to be, indeed, an improvement, and the more we have investigated the matter, the more we have become convinced of its utility. Thousands of *healthy—sound—beautiful* teeth have been sacrificed, without remorse, by our professional predecessors, in their now obsolete practice of making their clasps as small as possible.

A case in point has just come under my notice; allow me to exhibit one of the victims to that erroneous custom of the dental dark ages. And would you believe it? the subject of this malpractice is not a venerable grandmother, she is an interesting young married lady; showing that the abominable practice of clasping cuspidati is yet rife in some parts of this continent. A few years have sufficed, in this case, to destroy, to all intents and purposes, one of the most important teeth in this patient's mouth, besides causing her exquisite sufferings whenever partaking of sweet or acid aliments, cold or warm drinks.

This illustration, if known to any conscientious dentist, would I am sure, deter him from constructing similar contrivances.

The effect of narrow clasps is likewise injurious upon bicuspids and molars. We well remember a case, which occurred some two years since, in which four molars and two bicuspids were destroyed.

In order to conceal them from sight, the clasps were fastened upon the second molars, and made of small, half round wire, running along the first molar and second bicuspid on either side of the mouth.

The plate was very narrow, and the whole weight of the apparatus devolved upon the back teeth. In a short time violent inflammation of the gums supervened wherever the wire touched; palliatives were resorted to, and the patient not being very sensitive, persisted in wearing the teeth.

In a few years, one of the second molars having fairly been drawn from its socket, dropped in the mouth, still clasped by the wire, and caused the patient to apply for advice. Upon examination, we found the roots of the five remaining teeth exposed to the very apex, the lingual side of the alveolar entirely absorbed, and each tooth had a groove as nicely cut into it as might have been done with a saw.

In order that the two preceding illustrations be not supposed to be extreme cases, and of unfrequent occurrence, we beg leave to allude to another, in which the two canine teeth were destroyed although the plate was large. Eighteen months sufficed to expose freely to view the nerves of both cuspidati. Another set of teeth was now made, with two more teeth, and clasped, as previously, to the nearest teeth, the first bicuspid on either side, and the result was similar.

Both this, and the artificial piece alluded to in the preceding illustration, were executed in a workmanlike manner, and evinced great mechanical skill, which, however, could not atone for want of judgment, and the pernicious effects of the small clasps.

Some three years ago we were consulted by an eastern lady, who a few years previous had a lateral incisor replaced by the present arrangement. Any respectable practitioner might be puzzled to tell in what manner such a contrivance could possibly be retained in the mouth. Still, it was tolerated there nearly three years, and particular attention was only called to it from extreme sensitiveness in the canine tooth adjoining.

Upon examination, we fouud this appendage wafting to the gentlest breeze caused by the breath of the fair patient; considerable *engorgement* of the gums above and around the adjoining cuspidatus, and a sort of ring beneath the loose edge of the gum, as often produced by tartar. Still we could not account, from the construction of the artificial tooth, how it was so tenaciously held in its clapper-like position.

However, the application of a scaler to the supposed ring of tartar, showed it to be metallic.

The dentist had cut the gordian knot. Unable to make the artificial tooth remain in the mouth, he had tied it fast to the next tooth by means of two turns of fine silver wire. The result was a complete exposure of the nerve, and consequently the loss of a valuable tooth, which it became our duty to replace.

These instances have multiplied to an infinite number, and are daily related to us by patients in all their details, losing nothing of their beauties by circulation, and thus:

"Mr. So-and-so is no better than he should be. He ruined that lady's teeth. He ought to be sued for damages. I would not have suffered him to treat me in such a manner."

There may or there may not be truth and justice in these complaints; the dentist may be ignorant, eager for gain, or unprincipled. But it also sometimes happens that the patient is the very cause of such mischief being inflicted, by beating down the dentist's price—wishing him to insert a set of teeth for less than actual cost of materials, if made in a professional manner.

In such a case, we can neither entertain sympathy for the sufferer, nor make any apology for the dental practitioner.

We are fully convinced that narrow clasps are injurious, and have not the least doubt that the larger number of the profession entertain the same opinion.

Of late years, respectable dentists have abandoned the practice of clasping with small, half round wire, and have made it a point to cut a clasp as broad as the tooth would permit. The size of plates has also increased; and under such modifications, we cannot say from actual experience, that teeth have been sacrificed, nor do we deem it likely. Still, it must be obvious that, in dispensing entirely with clasps, when the nature of the case admits of it, we eradicate even the shadow of a doubt, in the patient's mind, as to the propriety and safety of inserting and wearing artificial teeth.—*Dent. News Letter.*

